# Challenges and opportunities for early career medical professionals in cardiovascular magnetic resonance (CMR) imaging: a white paper from the Society for Cardiovascular Magnetic Resonance

**DOI:** 10.1186/s12968-023-00968-3

**Published:** 2023-11-16

**Authors:** Purvi Parwani, Tiffany Chen, Bradley Allen, Kimberly Kallianos, Ming-Yen Ng, Rebecca Kozor, Olukayode O. Aremu, Kanwal M. Farooqi, Aurelio Secinaro, Fabrizio Ricci, Sarah Moharem-Elgamal, Gabriela Liberato, Akhil Narang, Vineeta Ojha, Chiara Bucciarelli Ducci, Sven Plein, Karen G. Ordovas

**Affiliations:** 1 Division of Cardiology, Department of Medicine, Loma Linda University Health, Loma Linda, CA USA; 2 Department of Clinical Medicine, Division of Cardiovascular Medicine, Hospital of the University of Pennsylvania, Philadelphia, PA USA; 3 Department of Radiology, Northwestern University, Chicago, IL USA; 4 Department of Radiology and Biomedical Imaging, University of California San Francisco, San Francisco, CA USA; 5 Department of Diagnostic Radiology, The University of Hong Kong, Hong Kong Special Administrative Region, China; 6 Department of Medical Imaging, The University of Hong Kong-Shenzhen Hospital, Shenzhen, China; 7 Cardiology Department, Royal North Shore Hospital and Faculty of Medicine and Health, University of Sydney, Sydney, Australia; 8 Division of Cardiology, Department of Medicine, University of Cape Town, Cape Town, South Africa; 9 Hatter Institute for Cardiovascular Research in Africa, Department of Medicine, University of Cape Town, Cape Town, South Africa; 10 Department of Pediatrics, Division of Cardiology, Columbia University Medical Center, New York, NY USA; 11 Advanced Cardiovascular Imaging Unit, Department of Imaging, Bambino Gesù Children’s Hospital, IRCCS, Rome, Italy; 12 Institute of Advanced Biomedical Technologies, Department of Neuroscience, Imaging and Clinical Sciences, “G. D’Annunzio” University, Chieti, Italy; 13 Cardiology Department, Liverpool Heart and Chest Hospital, Liverpool, UK; 14 Cardiology Department, National Heart Institute, Giza, Egypt; 15 Diagnostic Imaging Department, Heart Institute (InCor), University of São Paulo Medical School, São Paulo, Brazil; 16 Department of Radiology, Sirio Libanês Hospital, São Paulo, Brazil; 17 Northwestern University Feinberg School of Medicine, Chicago, IL USA; 18 Department of Radiology, Mahajan Imaging, Pushpawati Singhania Research Institute (PSRI), New Delhi, India; 19 Department of Cardiology, Royal Brompton and Harefield Hospitals, London, UK; 20 Department of Biomedical Imaging Science, University of Leeds and Kings College London, London, UK; 21 Department of Radiology, University of Washington, Seattle, WA USA; 22 Clinical Research Center, Department of Clinical Sciences, Lund University, Malmö, Sweden

**Keywords:** CMR, Imaging, Early career, Cardiology, Radiology

## Abstract

The early career professionals in the field of Cardiovascular Magnetic Resonance (CMR) face unique challenges and hurdles while establishing their careers in the field. The Society for Cardiovascular Magnetic Resonance (SCMR) has expanded the role of the early career section within the society to foster the careers of future CMR leaders. This paper aims to describe the obstacles and available opportunities for the early career CMR professionals worldwide. Societal opportunities and actions targeted at the professional advancement of the early career CMR imagers are needed to ensure continuous growth of CMR as an imaging modality globally.

## Introduction

Early Career (EC) is typically defined as those within seven years of completion of a cardiology or radiology training program [1]. Like any other early career professionals (ECP), imaging ECP often struggle to establish themselves in the field of cardiovascular magnetic resonance (CMR).

The Society for Cardiovascular Magnetic Resonance (SCMR) has recently expanded the structure of the EC Section and appointed the Chair of the Section as a member of the Board of Trustees, with the goal of increasing the impact of ECP within the society, understanding their specific challenges and developing targeted actions to address them. These include mentoring and professional development initiatives to foster the careers of the future CMR leaders.

**Fig. 1:**
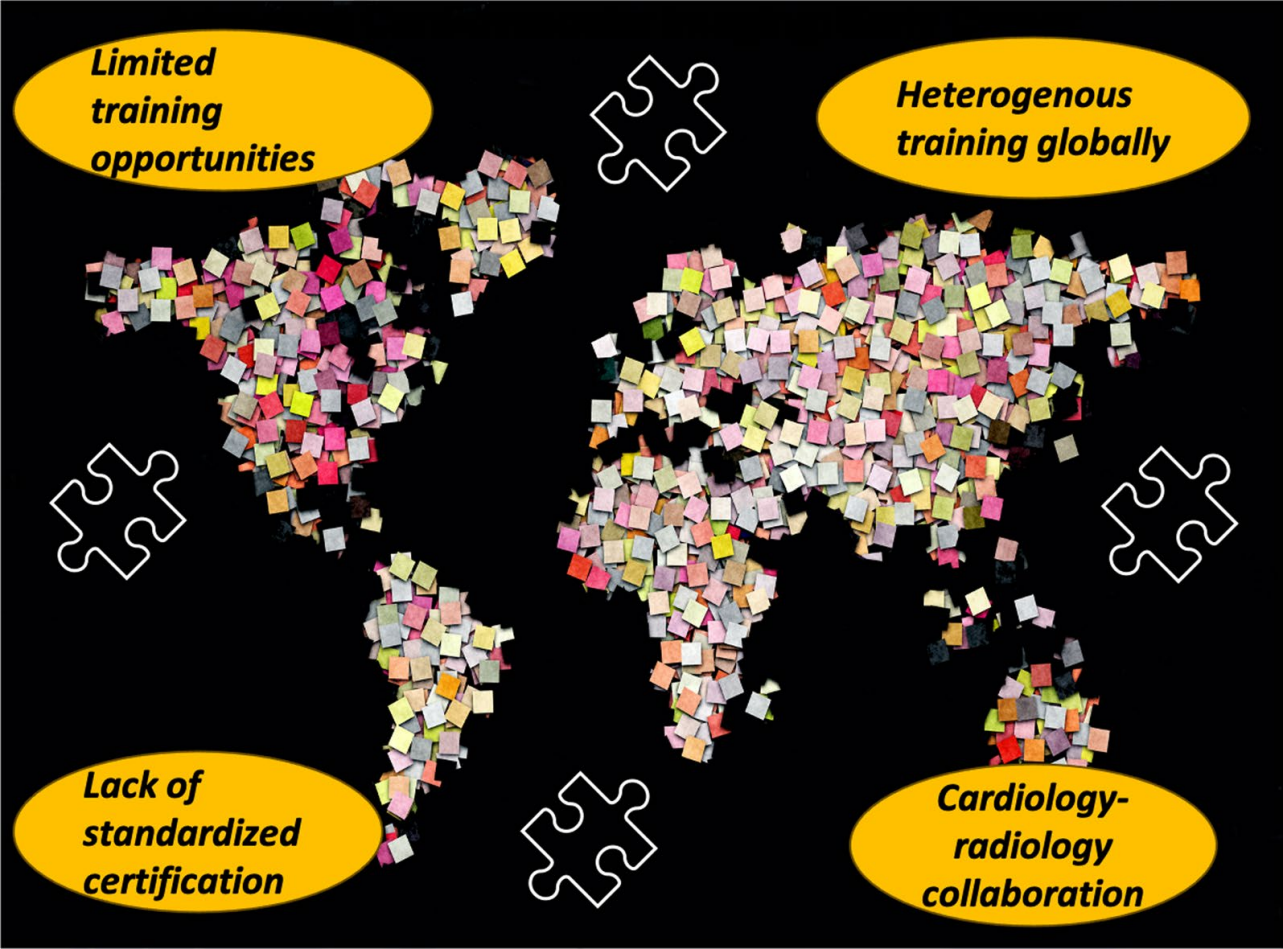
Central Illustration: Challenges of Early Career Professionals Worldwide

CMR is a clinically impactful and cost-effective diagnostic tool for various indications that include myocardial viability, stress perfusion, heart failure, hypertrophic cardiomyopathy, arrhythmia, congenital heart disease, valve disease, and aortic and other vascular pathologies [2–4]. In the past 30 years, CMR has emerged as a powerful non-invasive imaging modality with an increasing amount of research reflected in the publications worldwide. The number of published papers featuring CMR has increased from 500 papers/ year in 1990 to almost 3,000 papers/ year in 2018 (Fig. 1). Due to the increasing CMR clinical indications and an increasing focus on efficient and well-defined protocols which require less time on the scanner and less direct involvement by the cardiac imaging specialists [5], the number of clinical CMR studies is increasing. In Canada, for example, CMR utilization increased ten-fold between 2002 and 2016 in patients with heart failure [6]. Anecdotally, the pattern of CMR orders by clinicians seem to correlate with training in CMR, which highlights a potential opportunity for further expanding CMR in clinical practice by ensuring adequate exposure during cardiology training. The increased utilization of CMR is supported by the incorporation of CMR in multi-specialty practice guidelines and appropriate use criteria for evaluation of ischemic heart disease, cardiomyopathy, valve disease, and heart rhythm disorders and other cardiovascular indications [7–9]. The advancement of the field of CMR has also evolved the scope of training over the last few decades [10]. There is an increased demand for highly qualified imagers within cardiology and radiology with specialized training in CMR across the world [11] (central illustration). As demand increases, ECPs across the world have faced unique challenges.

This document was prepared by the SCMR EC section with input from the SCMR executive committee. It aims to describe the obstacles, challenges and available opportunities for ECP in CMR worldwide. The overall goal is to motivate actions to increase the engagement of early career professionals in the field of CMR.

## Opportunities and challenges for EC CMR professionals across the world

### North America

The advancement in the field of CMR and increased need for CMR imagers in North America has not been proportional to the increase in the number of trainees pursuing additional CMR expertise within radiology or cardiology. Imaging councils of various international societies have recognized a potential scarcity in the cardiovascular workforce to meet the demands of patients with cardiovascular (CV) disease [12]. Spilberg et al. [13]. found that only 7% of American College of Radiology (ACR) accredited sites in Massachusetts have further accreditation in CMR [13], indicating the potential need for more qualified CMR readers. A recent analysis found that amongst 33,090 practicing US radiologists, based on billed work relative value units, only 5.9% practiced as subspecialized cardiothoracic imagers. In this study, 10.3% of academic hospital radiologists were cardiothoracic imagers, while only 3.7% of nonacademic radiologists subspecialized in cardiothoracic imaging [14].

Although practicing CMR can afford a wealth of opportunities for early career cardiologists in the United States, specific challenges exist in terms of obtaining training and establishing a career in CMR. Most trainees have limited exposure to CMR in the context of a general cardiology fellowship, though few may attain competency for independent CMR practice (i.e. Core Cardiology Training Symposium [COCATS] level II) [10, 15]. In most cases, and for those interested in multi-modality imaging, proficiency in CMR (COCATS level II or III) requires dedicated training in the form of an advanced cardiovascular imaging (ACVI) fellowship. Currently, there are only 64 ACVI training programs in the United States [16], some funded through philanthropy and other mechanisms that are difficult to sustain [17]. The lack of formal accreditation by the Accreditation Council for Graduate Medical Education (ACGME) for such a subspecialty fellowship has resulted in widely heterogeneous and non-standardized training experiences, dependent on resource availability and expertise of individual programs. For instance, experience in stress perfusion or congenital CMR may be limited at individual centers. Despite standards for CMR proficiency set by the SCMR [18], training experiences vary in terms of breadth of training in other imaging modalities and duration of training (typically 1–2 years). Additionally, not all ACVI programs are CMR-centric or involve radiology faculty, who may be more experienced in physics and technical aspects [17]. Cardiologists often have minimal exposure to the physics and technical aspects of CMR during a general fellowship, and it is not until advanced training in CMR that they can dive deep into CMR image acquisition and interpretation. Whilst radiologists often have years of exposure during residency/fellowship to MR sequences, cardiologists have a much more compressed timeline that many have described as “learning a new language.” After advanced fellowship training, Certification Board of Cardiovascular Magnetic Resonance (CBCMR) board certification incurs another financial burden in addition to the panoply of other costs necessary for credentialing (such as board certification for general cardiology, echocardiography, and other imaging modalities). Establishing a career in CMR also presents several challenges to the cardiologists. In addition to the usual challenges of finding the right job, specific factors related to the inequities in the reimbursement system in the United States exist. Physician reimbursement for CMR, in terms of relative value units (RVUs), is not commensurate with the time necessary for interpretation and reporting [19]. Therefore, an imaging cardiologist or radiologist with RVU-based compensation may be pressured to perform other, higher RVU-generating, clinical activities that limit availability to read CMR.

Furthermore, due to clinical workloads, cardiologists may not always be present at the scanner or available in real-time for troubleshooting, artifact resolution, and optimization. In a survey of 180 ACVI graduates, 87% practice a blend of clinical cardiology and imaging, while only 8% practice cardiac imaging only [20]. Less than half (48%) surveyed found it easy to find a job that “encompassed the imaging elements” they trained for [20]. EC cardiologists in the United States seeking to build CMR programs may have to contend with the financial challenges of operating within the constraints of a complex payment system, which has reduced reimbursement for technical fees [19]. Competing interests and priorities may hinder imaging cardiologists establishing CMR expertise at an institution where equipment is owned or managed by radiology and utilized for non-cardiac imaging services. Scanner time for cardiac examinations may require negotiation, and it may not be feasible to have dedicated cardiac MR scanners or technologists. Some institutions have implemented joint reading with revenue sharing [11]. Collaboration between cardiology and radiology is required to foster CMR program growth, enrich research and educational opportunities, and promote the advancement of early-career faculty, [11] but may remain a challenge, depending on leadership dynamics. Thus, the climate of cardiology-radiology relations can influence the scope of practice, job satisfaction, and research productivity of early career CMR imagers.

For radiologists, most academic training programs have CMR included as part of the radiology training curriculum, and these experiences can vary from high volume clinical exposure for 3–4 months throughout the four-year training program down to 2 weeks for the entire training experience. The clinical experience during training can range from reviewing cases together with a radiology and/or cardiology attending physician trained in CMR and may advance to supervised interpretation of emergent studies, independent post-processing, and protocoling studies. Based on a trainee’s exposure, comfort level with interpreting these studies in practice as well as interest in pursuing fellowship training in cardiovascular imaging can vary widely. However, it is important to notice that all board-certified radiologists in the US have achieved competency for performance and interpretation of CMR since the method is a required component of the American Board of Radiology examination. Important challenges radiology trainees face in CMR include becoming comfortable with cardiac physiology and pathophysiology and recognizing how cardiac diseases manifest on standard and advanced CMR sequences.

Radiologists acquire advanced cardiac imaging subspecialty training either through a cardiothoracic imaging fellowship, where the trainee usually obtains expertise in both cardiovascular and chest imaging, or a dedicated cardiovascular imaging fellowship. Cardiothoracic fellowships are a common route for US radiologists to obtain advanced training in CMR, likely because the training in both cardiac and chest imaging helps broaden their skillset and improve their overall opportunities in the job market. Depending on the fellowship, trainees usually graduate with level III equivalent CMR certification. In some settings, other fellowships such as magnetic resonance imaging, body imaging or mini fellowships are being offered by societies such as the American College of Radiology (ACR) or the SCMR. In most settings, cardiac imaging remains a relatively small percentage of overall radiology imaging volume, while chest imaging (chest radiographs, chest CT) continues to expand. Therefore, radiologists who practice CMR generally also have subspeciality training in at least one additional radiology subspecialty. Relatedly, the number of cardiothoracic imagers in radiology is small. A survey of 353 US radiology trainees found that only 4.5% planned to pursue fellowship training in either cardiac or chest imaging, which was one of the lowest subspeciality totals in this cohort [21].

Specific qualifications are needed for radiologists to interpret CMR examinations, which are outlined in the ACR–North American Society of Cardiovascular Imaging (NASCI)– Society of Pediatric Radiology (SPR) practice parameter for the performance and interpretation of cardiac magnetic resonance imaging guidelines [22]. In addition to baseline expertise in the general principles of MRI, radiologists interpreting CMR must either (1) have received specific CMR training in an ACGME accredited radiology residency program or (2) have completed at least 30 h of continuing medical education credits in CMR, in addition to interpretation and/or supervised review of at least 50 CMR examinations in the preceding 36 months in both scenarios. Radiologists without prior expertise in the general principles of MRI require more extensive CMR training. This includes 200 h of continuing medical education credits including a focus on both general and cardiac MRI in addition to supervision and interpretation of at least 150 MRI examinations, including 50 CMR, in the preceding 36 months. Radiologists desiring to interpret stress CMR examinations must maintain Advanced Cardiac Life Support (ACLS) certification in addition to possessing knowledge on the administration, risks, and contraindications relevant to the agents used in pharmacologic stress testing. (Central illustration).

### Europe

The technical advances made in the late 1990s allowed the increased clinical application of CMR to spread in Europe to everyday practice. However, despite increased delivery of CMR, there is wide variability in CMR services and volumes of scans between and within European countries with the UK and Germany having the highest activity [23, 24]. Training opportunities at large CMR centers with their own dedicated scanners are very attractive and competitive to European cardiology and cardiac radiology trainees seeking fellowships to train in this subspecialty. A directory of CMR centers with training fellowships in Europe can be found at the European Society of Cardiology website [25].

In Europe, CMR training may be incorporated as a part of subspecialty training in cardiology or radiology depending on the subspecialty interest of the trainee, while others may seek a postgraduate doctorate degree to do research and learn more about CMR. However, many will pursue a dedicated fellowship during or after completing their specialty training, particularly those who wish to be an educator, leader and supervisor in a CMR unit. In addition to national societies, the European Association of Cardiovascular Imaging (EACVI) and European Society of Cardiovascular Radiology (ESCR), offer training and research grant opportunities for both cardiologists and radiologists to level-up their diagnostic skills, acquire knowledge, expertise and competencies for running and delivering new CMR services [26, 27].

Two certification processes with different criteria for CMR are available in Europe—a dedicated CMR certification program from the EACVI for cardiologists and radiologists or the Cardiovascular Radiology Diploma for CMR and CT offered by ESCR for radiologists [28, 29]. EACVI Level 3 certification requires: (1) physicians to have successfully passed the EACVI CMR exam, (2) spend at least 12 months of full-time training in CMR under a CMR Level 3 certified expert, (3) submit a logbook of 300 cases, (4) to have completed 50 h of CME specifically in CMR, (5) provide evidence of peer recognition as outlined on the EACVI website (https://www.escardio.org/Education/Career-Development/Certification/Cardiovascular-Magnetic-Resonance). European Board of Cardiovascular Radiology (EBCR) Diploma requirements include (1) RIS statistic printout of CMR and cardiac CT cases, (2) logbook with minimum of: 150 live-cases or 300 data-base-cases cardiac CT studies, 100 live-cases or 300 data-base-cases cardiac MR studies, 100 non-coronary vascular CT cases and 50 vascular MR cases, (3) to contribute to the ESCR MR/CT Registry Certificate with a total number of MR and CT cases submitted, (4) a letter of support from the head of department/program director is required, (5) at least 50 CME credits in cardiovascular radiology, recognized by ESCR.

Neither certification process is compulsory for a regulatory certificate to practice in Europe, but they both promote a European standard for CMR competency, acquisition and reporting and increase credibility, particularly when searching for job opportunities.

In many European countries, leading academic and clinical institutions have established clinical, training and research CMR programs and perform more than 1000 studies per year, while many other centers barely attain 300 clinical scans per year [30] According to the multi-national European cardiovascular magnetic resonance (EuroCMR) registry [23], CMR case reading and reporting was undertaken by cardiologists, a team of cardiologists and radiologists, or radiologists alone in 70.7%, 26.7% and 2.6% of cases, respectively. The British Society of Cardiovascular Magnetic Resonance (BSCMR) national survey performed in UK in 2019 showed that from the 82 CMR responding centers, there were 351 consultants reporting CMRs − 230 (66%) were cardiologists and 121 (34%) radiologists [24]. In the European MR/CT registry of the ESCR [31] and in the Italian CMR survey led by the Working Group of the Cardiac Radiology Section of the SIRM (Società Italiana di Radiologia Medica) [32], both were characterized by predominant radiological involvement at participating sites—at least 80% of exams were read and reported by radiologists, plus 17–19% of cases performed in consensus with cardiologists. However, these surveys were sent to predominantly radiologists from both societies’ mailing lists. Notably, the right to report and sign off clinical CMR studies in individual countries remains defined by different national laws and regulations, with varying implications across European countries, at times significantly hampering sustainable EC pathways in both clinical and research settings. Overall, in Europe, there are various pathways of training and certification processes to promote competency and practice models for delivering clinical CMR services led by cardiologists, radiologists or jointly, both in the public and private sectors. Overcoming this segregation and providing equitable access to CMR for both cardiology and radiology trainees will be critical for the ongoing growth of CMR in Europe.

### Australia/NZ

CMR is performed and reported by cardiologists and radiologists in Australia and New Zealand (ANZ), most commonly associated with tertiary referral hospitals and university hospitals. However, there is also a proportion done in the private sector.

The biggest obstacle for CMR in Australia is the upfront cost and accessibility of CMR scans. This issue revolves around limited reimbursement from the national healthcare system, Medicare. Currently there are only four restricted indications for reimbursement; congenital heart disease, aortic pathology, cardiac masses, and arrhythmogenic right ventricular cardiomyopathy (ARVC) (the latter only added in 2019). Thus, the common indications of heart failure, cardiomyopathy other than ARVC, myocarditis and coronary disease are not covered and result in patients paying out-of-pocket or referrers choosing alternative investigations (e.g. stress echocardiography and cardiac CT which are prevalent in Australia). This has led to CMR being only a small part of cardiology and radiology, and physicians consequently do not solely specialize in CMR, but rather, practice in conjunction with other interests.

CMR is not formally included in cardiology or radiology specialty training. To gain formal experience in CMR, one needs to do a fellowship, either locally or internationally. There are multiple sites in Australia that offer CMR fellowships, but many cardiologists and radiologists interested in the field seek training overseas. In 2019, a formal certification process in CMR was introduced in ANZ, hence now early career professionals and trainee physicians interested in CMR need to meet these requirements. Traveling overseas to gain the necessary experience can put financial and time pressures on ECP, especially if they are self-funded or supported by a low-paying grant.

Despite the challenges, there is growing interest and excitement for CMR in ANZ, driven by the rising stars and ECPs alongside the many highly experienced and renowned senior academics and physicians. There is an ANZ Working Group in CMR that strives to provide CMR education and advocacy. It organizes educational activities like the annual *CMR Australia* symposium, hosts international speakers, and supports online learning for accreditation purposes. Because ANZ is geographically large, attending such meetings can be difficult for trainees and early career physicians particularly from regional and remote areas.

CMR is recognized as an increasingly important part of cardiovascular research. However, in ANZ the remuneration for CMR is less than for other clinical work and often challenging to obtain if it relies on an external grant to ‘buy time’ out of clinical work. This can be a disincentive for early career researchers and causes job insecurity. One solution is to diversify work (public, private, teaching, research) to ensure stable and sustainable career pathways. Despite the challenges, there is huge potential and enthusiasm in ANZ for CMR research, as well as the development of engagement and advocacy strategies, and to develop programs for early career professionals to establish partnerships with national and international leaders in the field, building networks that will benefit ANZ and CMR globally.

### Latin America

In Latin America, there has been an increase in CMR exams since the 2000s, particularly in Brazil following the publication of the Cardiovascular Imaging Guideline in 2006 [33]. Since then, in the face of growing scientific evidence and new technological developments, CMR has been widely disseminated.

According to data from the SCMR, it is estimated that between 33,000 and 55,000 CMR exams/ year are currently performed in Latin America, mainly in Brazil, which has the largest number of centers, followed by Cuba, Chile, Mexico, and Argentina. Also, the number of SCMR members from each country shows similarities with these data, with members from Cuba the most representative, followed by Brazil and Chile [34].

Despite all technological advances, most centers in Latin America still use only basic CMR acquisition protocols, such as cine, perfusion, and delayed enhancement sequences. Due to the high cost of purchasing scanners with new sequences, only a few centers (mostly research) have capabilities for T1 and T2 mapping, for example. Although on one hand there is a considerable irregular distribution of these latest CMR sequences in Latin America territory comparing to North America and Europe, on the other hand, there is still a void to explore and develop new technologies with the most prevalent diseases in Latin America.

Most CMR specialists in Latin America are cardiologists, followed by radiologists. In general, additional training (after the radiology or cardiology fellowship) is required for Cardiovascular Imaging. The dedicated training in the form of an advanced cardiovascular imaging program (cardiovascular CT and MR) lasts between 12 and 24 months in Brazil. Radiologists can have this additional cardiovascular training as a cardiothoracic program. Robust training programs have been developed in Argentina, Chile, Colombia, Mexico and Peru.

The training centers follow the recommendations of the SCMR guidelines [35], and although capable to provide full board level certification and collaboration with the international CMR community, in some countries there is still no specific CMR certification by the local medical societies [36].

### Asia

In Asia, each country faces particular opportunities and challenges due to the diverse socioeconomic, cultural and healthcare system models. There are many challenges facing EC cardiologists and radiologists in Asia wanting to learn, establish or promote CMR services.

Some Asian countries have proportionally lower number of doctors per 10,000 population compared to their North American and European counterparts, which inhibits sub-specialization in CMR [37]. In these situations, radiologists and cardiologists do not have the opportunity to dedicate time to a sub-specialty focus such as CMR as they are expected to cover a wide variety of radiology or cardiology specific roles. Given the contemporary nature of CMR as an imaging modality, senior cardiologists or radiologists may have minimal CMR training and thus limited capability to transfer CMR knowledge and skills to the upcoming generation. Basic CMR knowledge is also not a training requirement in some of the Asian national radiological or cardiology societies/ colleges. The complexity of CMR and the requirement for sufficient training in CMR to establish and run a high performing service, further prevents CMR from establishing itself in the clinical realm. The option of attending high-quality CMR workshops is also hampered by cost which maybe beyond the reach of doctors’ salaries in some of the poorer Asian countries. In addition, scientific publications related to CMR are primarily in English and online CMR learning sites are commonly in English only. This presents a hurdle for educational outreach and promoting research collaboration with overseas non-English speaking national centers. Furthermore, in Asian countries like China, there is lack of availability of adenosine or regadenoson for stress CMR, which limits the exposure of trainees to this stress modality [38].

CMR educational opportunities are now being provided in many different formats (see table below). The SCMR has provided a webinar series on CMR basics delivered entirely in Chinese (https://scmr.peachnewmedia.com/store/seminar/seminar.php?seminar=184157). The Asian Society of Cardiovascular Imaging (ASCI) delivers CMR knowledge to the medical community via its annual scientific meetings combined with SCMR sessions [39]. Singapore has established a 6 to 12-month CMR fellowship program with SCMR for local and other Asian trainees to obtain valuable CMR knowledge and skills [40]. CMR workshops are held annually in many Asian countries like India in collaboration with SCMR which indicates the growing use and interest of CMR in Asia. At centers like the University of Hong Kong, free online CMR lectures, animations and learning cases with Chinese translations was launched in 2021 in collaboration with SCMR to combat language and financial barriers (https://scmr.peachnewmedia.com/store/seminar/seminar.php?seminar=182954).

With growing interest in CMR in Asia, fellowship training is being sought by Asian radiologists and cardiologists. Countries like India have their own subspecialization courses in cardiovascular radiology, albeit only in a few major cardiac centres. Furthermore, Asian radiologists and cardiologists have been returning from overseas fellowship training which has resulted in drastic increase in the use of CMR in many hospitals. This has allowed for more research and grant opportunities for cardiologists and radiologists, as well as the opportunity to conduct CMR research specifically on Asian patients with cardiovascular issues unique to the region.

### Africa

Africa is a large continent, comprising of 54 countries, and a total population of 1.4 billion people. Many of the countries in Africa are classified as low- or middle-income countries in the Development Assistance Committee list of official development assistance recipients. CMR is not widely available on the continent and is mainly concentrated in large urban centres, where it is almost exclusively read by radiologists. The majority of MRI scanners are located within the private sector with relatively few in the public sector and academic centres. South Africa is the only country with over 20 centres now performing high-quality CMR imaging on a regular basis. The largest CMR fellowship programs in Africa can be found at the University of Cape Town in South Africa, or Aswan Heart centre in Egypt.

Beyond limited availability of scanners and expertise in CMR, other important challenges for training in the technique include, low volumes of scans performed in many centres, technologists without training in CMR, and a referral bias by cardiologists preferring invasive angiography or nuclear cardiology tests to complement echocardiographic imaging. Funding for CMR also remains a big challenge with few people who can afford out-of-pocket payments, and many healthcare funders still seeing CMR as a “nice to have” rather than a “must have” medical investigation [41]. In West Africa, with a combined population of more than 350 million, infrastructural and maintenance limitations pose a major obstacle to utilization of high field MRI equipment, with very few MRI scanners for the population [42]. Another great challenge is the poor collaboration between public and private sectors and ongoing “turf” and ownership battles of CMR service between cardiology and radiology.

Several important opportunities exist. First, there is a critical need for training platforms for clinicians, technologists and students in CMR. Established in 2016 to promote CMR practice, education, research and advocacy on the African continent, the annual Cardiovascular Magnetic Resonance Congress of South Africa (SACMR) was the first platform that brings together the cardiologists, radiologists, technologists, physicists, biomedical engineers and student trainees with an interest in CMR [43]. In addition, both at the Cape Universities Body Imaging Centre at the University of Cape Town and at Aswan Heart Centre, offer hands-on CMR courses, attended by delegates from all over the continent. These courses are an important opportunity for collaboration between cardiologists and radiologists and in building a new generation of scientists and clinicians with grounding and interest in CMR.

### Pediatric CMR

In the past decade, the need for pediatric CMR (pCMR) experts has increased. This is in part due to a significant role for pCMR having been identified for the clinical management of patients with congenital heart disease (CHD) [44, 45]. Nonetheless, multiple obstacles remain for the ECP growth. In the United States (US), application to an advanced imaging fellowship for additional training in pCMR is made during the third year of pediatric cardiology fellowship or during general CMR training for the radiologist. In most of the European countries, additional pCMR mentoring programs can be undertaken during Cardiology and Radiology qualifications. There remains some lack of uniformity in pCMR training and EC programs among European countries. In choosing a desirable training program, factors which aid in selection include pCMR curriculum, learning objectives, fellowship location, volume and complexity of cases and experience of teaching faculty. In the US, there are approximately 20 pediatric advanced imaging training programs for cardiologist. For radiologists, generally the training is provided during advanced cardiac or cardiothoracic or pediatric radiology fellowship. Guidelines for training in CMR do not specify the specific numbers of pediatric cases to be qualified to read congenital cases however in level II and Level III CMR certification, exposure to enough adult and pediatric cases is encouraged. Information on pCMR training programs is available on both the SCMR and the Society of Pediatric Cardiology Training Program Directors websites [46, 47]. Recommendations have been formulated to describe program resources and environment required for appropriate non-invasive imaging fellowship training [48]. In Europe, training programs are increasingly promoted and supported by Imaging Societies. For instance, the EACVI offers a process for certification of individuals in congenital and pCMR [49]. There are about 20 European centers which have Level 3 certification from EACVI in pCMR for CHD [50].

Due to the limited number of pCMR training programs, partiality to internal candidates and geographic constraints, the application process can be challenging and competitive. Identifying a viable option for a pCMR fellowship position as early as possible and becoming familiar with the program’s pCMR faculty members is key for a successful application. Within a few months of starting a program, however, the search for a job begins. Considerations for choosing a suitable position include program size and location, volume of pCMR, percentage of designated clinical versus research time, access to mentorship and compatibility with potential future colleagues. Although there are annual variations, pCMR positions in the US are typically limited, given that smaller Pediatric Cardiology programs usually only need one pCMR specialist on staff. The same is true for programs in Europe, where pCMR vacancies are classically constrained due to limited Pediatric Cardiology volume. Physicians are therefore sometimes discouraged from seeking additional training and practicing in this field. Practical challenges which can arise soon after starting a new pCMR position include adjustment to a new scanner, and to the system used for post-processing and reporting. In addition, one must adapt to the collaborative structure in place between Cardiology and Radiology for the interpretation and billing of pCMR. This can be challenging in the setting of being the new member of an imaging team but is essential to form collegial professional relationships. pCMR is different from other imaging studies in the time which needs to be dedicated to its performance and post-processing. It is important to ensure that one’s institution is cognizant of this fact so that time can be allotted in the schedule for appropriate reporting. There needs to be communication with institutional administration regarding this time commitment, with a system set in place to avoid penalties associated with potentially lower annual relative value units (RVU) compared to other imaging specialists in the US. Similarly, European pCMR is still particularly cost ineffective. Despite significant time investment, particularly for exams performed under general anesthesia, these studies offer disproportionately low reimbursements and introduces other challenges like anesthesia availability, additional cost, longer room turnover time. Interestingly, access to future technologies such as faster sequences, combined with artificial intelligence algorithms may improve imaging workflows with clear benefits for pCMR service sustainability [51].

There are a number of specific SCMR membership opportunities that are available to connect with colleagues in the pCMR community, including the Pediatric and Congenital Heart Disease Section which consists of multiple subcommittees and special interest groups. European pCMR networking is promoted by various imaging societies including the Association for European Pediatric and Congenital Cardiology (AEPC), EACVI, and The European Society of Pediatric Radiology (ESPR), some of which are very active with dedicated sections, taskforces and working groups. SCMR recently launched its first Pediatric and Congenital CMR advanced course with hands-on sessions (analysis and scanning) which will become a regular educational event (https://scmr.org/events/EventDetails.aspx?id=1733613&hhSearchTerms=%22munich%22).

## Conclusion

Although there is a growing demand for CMR professionals worldwide, ECPs face significant challenges while establishing their careers in the field. National and international societal opportunities and interventions are required to facilitate the advancement of EC CMR imagers, which will further enhance the growth of CMR as an imaging modality worldwide. To find out more about CMR fellowships available internationally, please consult the SCMR website (https://scmr.org/page/Fellowships).

## Data Availability

Not applicable since this is a white paper.

## Abbreviations

ACVI: Advanced Cardiovascular Imaging
ACGME: Accreditation Council for Graduate Medical Education
ARVC: Arrhythmogenic right ventricular cardiomyopathy
ASCI: Asian Society of Cardiovascular Imaging
BSCMR: British Society of Cardiovascular Magnetic Resonance
CBCMR: Certification Board of Cardiovascular Magnetic Resonance
CHD: Congenital heart disease
CMR: Cardiovascular Magnetic Resonance
COCATS: Core Cardiology Training Symposium
CV: Cardiovascular
EACVI: European Association of Cardiovascular Imaging
ECP: Early Career Professional
ESCR: European Society of Cardiovascular Radiology
ESPR: The European Society of Pediatric Radiology
pCMR: Pediatric CMR
SACMR: Cardiovascular Magnetic Resonance Congress of South Africa
SCMR: Society for Cardiovascular Magnetic Resonance
SIRM: Società Italiana di Radiologia Medica

## Declarations

### Peer-review

This work represents an official publication of the SCMR and was approved by the SCMR Executive Committee. It did not undergo *JCMR* peer-review.
